# Spatial regulation of AMPK signaling revealed by a sensitive kinase activity reporter

**DOI:** 10.1038/s41467-022-31190-x

**Published:** 2022-07-05

**Authors:** Danielle L. Schmitt, Stephanie D. Curtis, Anne C. Lyons, Jin-fan Zhang, Mingyuan Chen, Catherine Y. He, Sohum Mehta, Reuben J. Shaw, Jin Zhang

**Affiliations:** 1 Department of Pharmacology, University of California San Diego, La Jolla, CA USA; 2 Molecular and Cell Biology Laboratory, The Salk Institute for Biological Studies, La Jolla, CA USA; 3 Department of Bioengineering, University of California San Diego, La Jolla, CA USA; 4 Department of Chemistry and Biochemistry, University of California San Diego, La Jolla, CA USA

**Keywords:** Fluorescence imaging, Nutrient signalling, Kinases, Biosensors, Fluorescent proteins

## Abstract

AMP-activated protein kinase (AMPK) is a master regulator of cellular energetics which coordinates metabolism by phosphorylating a plethora of substrates throughout the cell. But how AMPK activity is regulated at different subcellular locations for precise spatiotemporal control over metabolism is unclear. Here we present a sensitive, single-fluorophore AMPK activity reporter (ExRai AMPKAR), which reveals distinct kinetic profiles of AMPK activity at the mitochondria, lysosome, and cytoplasm. Genetic deletion of the canonical upstream kinase liver kinase B1 (LKB1) results in slower AMPK activity at lysosomes but does not affect the response amplitude at lysosomes or mitochondria, in sharp contrast to the necessity of LKB1 for maximal cytoplasmic AMPK activity. We further identify a mechanism for AMPK activity in the nucleus, which results from cytoplasmic to nuclear shuttling of AMPK. Thus, ExRai AMPKAR enables illumination of the complex subcellular regulation of AMPK signaling.

## Abstract (web summary)

AMP activated protein kinase (AMPK) is a master regulator of cellular metabolism, but how AMPK activity is spatiotemporally regulated remains unclear. Here, Schmitt et al develop a sensitive biosensor for AMPK, which they use to uncover mechanisms for AMPK activity in the lysosome and nucleus.

## Introduction

AMP-activated protein kinase (AMPK) is a ubiquitously expressed heterotrimeric protein in mammals, composed of one of two α kinase subunits, one of two β regulatory subunits, and one of three γ nucleotide-binding subunits^1^. Allosteric activation of AMPK is achieved through binding of adenine nucleotides to the γ subunit or small molecules to the allosteric drug and metabolite (ADAM) site at the interface between α and β subunits^2,3^. AMPK is regulated by several upstream kinases, predominantly liver kinase B1 (LKB1)^4,5^ and calcium/calmodulin protein kinase kinase 2 (CaMKK2)^6^, to control metabolic processes including glycolysis, lipid and protein biosynthesis, mitochondrial biogenesis, and gene expression^7,8^.

How AMPK senses different stimuli and relays diverse signals to downstream components in various subcellular locations with high specificity has remained elusive. An emerging view is that compartmentalized AMPK signaling enables specificity toward downstream effectors^9,10^. Signaling complexes containing AMPK are found at different subcellular locations, including lysosomes, mitochondria, endoplasmic reticulum, and the nucleus^7^. Recent studies found that lysosomal pools of AMPK are preferentially activated by glucose starvation, whereas more severe metabolic stressors like glutamine starvation or pharmacological stimulation are required to activate cytoplasmic and mitochondrial pools of AMPK, due to specific assembly of lysosomal AMPK-activating signaling complexes^11^. However, our understanding of the regulation of AMPK signaling at different subcellular locations is still limited, and AMPK regulation in specific locations remains controversial^9,10^.

Genetically encoded reporters are powerful tools for interrogating the precise spatiotemporal regulation of signaling pathways^12,13^. Addition of localization tags for cellular organelles and compartments to kinase activity reporters enables subcellular resolution of kinase activities in single cells, allowing for the elucidation of compartment-specific signaling mechanisms^14,15^. This approach enabled us to profile subcellular AMPK activity using a Förster Resonance Energy Transfer (FRET)-based AMPK activity reporter^16^. However, the dynamic range of the current FRET-based AMPK activity reporters limited our ability to fully distinguish the dynamics and regulation of spatiotemporal AMPK activity^15–19^.

In this work, we develop a single-fluorophore excitation-ratiometric AMPK activity reporter (ExRai AMPKAR), which reports endogenous AMPK activity in living cells with high sensitivity. Using our reporter, we profile spatiotemporal AMPK activity and find spatially defined roles for LKB1 in regulating cytoplasmic, lysosomal, and mitochondrial AMPK activity. Finally, we measure nuclear AMPK activity with ExRai AMPKAR and identify a regulatory mechanism for  nuclear AMPK activity in living cells. This work provides a better understanding of spatiotemporal AMPK activity, which is critical for the diverse signaling profile of AMPK throughout the cell.

## Results

### Development of an excitation-ratiometric AMPK activity reporter

**Fig. 1:**
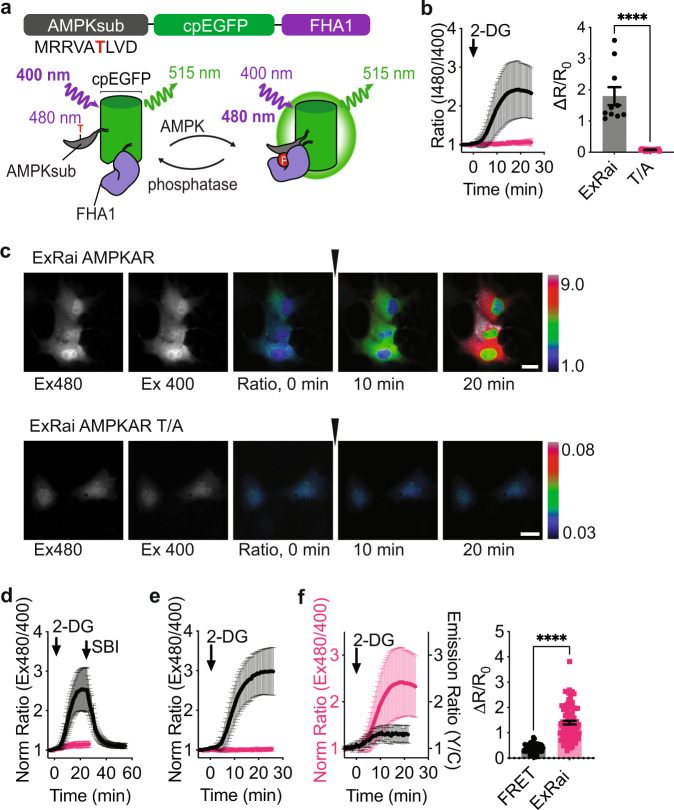
Development and characterization of ExRai AMPKAR. **a** Design and domain structure of ExRai AMPKAR. Threonine phosphorylated by AMPK denoted in red. **b** Average response of ExRai AMPKAR (black, *n* = 10 cells from three experiments), and ExRai AMPKAR T/A (pink, *n* = 17 cells from three experiments) to 2-DG (40 mM) stimulation in Cos7 cells along with maximum ratio change (****p = 3.05 × 10^−8^, unpaired *t*-test, two-tailed). **c** Representative images of ExRai AMPKAR (top) and ExRai AMPKAR T/A (bottom) in Cos7 cells treated with 2-DG (40 mM) at the indicated time. **d** Average response of ExRai AMPKAR to AMPK stimulation by 2-DG (40 mM) followed by SBI-0206965 (SBI, 30 µM, black, *n* = 35 cells from three experiments) or after pretreatment with SBI-0206965 in HEK293T cells (pink, *n* = 43 cells from four experiments). **e** Average response of ExRai AMPKAR in WT MEFs (black, *n* = 31 cells from four experiments) and AMPKα KO MEFs (pink, *n* = 23 cells from four experiments) treated with 2-DG (40 mM). **f** Response of ExRai AMPKAR (pink, *n* = 90 cells from five experiments) and FRET-based AMPK reporter ABKAR (black trace, *n* = 25 cells from two experiments) in HEK293T cells treated with 2-DG (40 mM), along with maximum ratio change (****p = 4.31 × 10^−19^, unpaired *t*-test, two-tailed). For all figures, time courses show the mean ± SD, dot plots show the mean ± SEM. Scale bars, 20 µm.

Building on our recent success in engineering single-fluorophore-based kinase activity reporters with high dynamic range^20,21^, we set out to develop a single-fluorophore AMPKAR. The modular design of biosensors enables coupling of different activity sensing units with readout-generating reporting units to create new biosensors^22,23^. We inserted a circularly permutated enhanced green fluorescent protein (cpEGFP), a widely used reporting unit^24^, between the two components of the AMPK activity sensing unit from a previous FRET-based AMPK biosensor^18^, which consists of an AMPK substrate domain^17^ and the phosphoamino acid-binding forkhead associated domain 1 (FHA1). Upon phosphorylation of the reporter and binding of the FHA1 domain to the phosphorylated AMPK substrate sequence, we expect to observe an increase in fluorescence emission with 480 nm excitation and a decrease with 400 nm excitation (Fig. 1a). The ratio of these two fluorescence intensities (Ex 480 nm/400 nm) can be used as an excitation ratiometric readout (R). Given that linkers at the junctions of the sensing unit and reporting unit are particularly important in determining the dynamic range of the reporter^25^, we set out to vary the two amino acids immediately flanking either side of cpEGFP to identify a linker combination that would result in a large excitation ratio change upon AMPK stimulation. The top five linkers from our previous screen to generate a high-performance excitation-ratiometric protein kinase A activity reporter were selected^21^, and we tested the linker variants in Cos7 cells stimulated with an AMPK activator, 2-deoxyglucose (2-DG, Supplementary Fig. 1a). From this linker screen, we found the linker combination FC/LL yielded the largest maximum ratio change (∆*R*/*R*_0_), and this variant was selected for further characterization.

Stimulation of AMPK using 2-DG in Cos7 cells expressing this reporter led to a large change in the excitation ratio (∆*R*/*R*_0_ = 1.80 ± 0.29; Fig. 1b, c and Supplementary Fig. 1b), whereas mutating the phosphorylation site threonine to alanine (T/A) to generate a phospho-null mutant resulted in a biosensor with minimal response (∆*R*/*R*_0_ = 0.075 ± 0.0064, *p* < 0.0001). We tested the reversibility of the response using the AMPK inhibitor SBI-0206965 (SBI)^26,27^. In HEK293T cells expressing the reporter construct, 2-DG-stimulated AMPK activity was rapidly suppressed by SBI-0206965 to near basal levels (*R* = 1.11 ± 0.011; Fig. 1d). Similarly, pretreatment with SBI-0206965 blocked the 2-DG-induced reporter response (∆*R*/*R*_0_ = 0.17 ± 0.011). We confirmed the ratio changes of ExRai AMPKAR are well correlated with phosphorylation of endogenous AMPK substrates (Supplementary Fig. 1c–e). To confirm the reporter responses are specific for AMPK activity, we expressed our biosensor in either wild-type or AMPKα1/2 knockout mouse embryonic fibroblasts (WT or AMPKα KO MEFs, Supplementary Fig. 1f)^28^. Treatment with SBI led to a decrease in the excitation ratio in WT MEFs, but not AMPKα KO MEFs (Supplementary Fig. 1g), suggesting presence of basal AMPK activity in WT MEFs. 2-DG stimulation induced large excitation-ratio increases in WT MEFs, whereas minimal responses were observed in AMPKα KO MEFs (Fig. 1e and Supplementary Fig. 1d), further demonstrating the specificity of the biosensor response. We observed a large variability in reporter response, and comparison of initial fluorescence (ex 400 nm) to ∆*R*/*R*_0_ did not show a strong correlation (Supplementary Fig. 1h). Previous studies have shown heterogeneous AMPK activity in a cell population when assessed at a single-cell level^29^, which is likely observed here with our biosensor.

Finally, we compared our new construct to our previous FRET-based reporter^18^. In HEK293T cells stimulated with 2-DG, the excitation-ratio change from our new construct (∆*R*/*R*_0_ =1.40 ± 0.072) was larger than the yellow/cyan emission ratio change from the FRET-based biosensor (∆*R*/*R*_0_ = 0.39 ± 0.024, *p* < 0.0001; Fig. 1f), which translated to a higher signal-to-noise ratio (SNR; FRET biosensor: 59.11 ± 6.46 vs. new construct: 228.1 ± 12.81, *p* < 0.0001, Supplementary Fig. 1i). We also estimated the Z-factor, a measurement of assay fitness^30^. From the average responses of these two reporters, the FRET-based biosensor had a Z-factor of 0.44, while our new construct had a Z-factor of 0.90, indicating better assay fitness. Thus, we have developed an AMPK activity reporter which we termed excitation-ratiometric AMPKAR (ExRai AMPKAR), for specific monitoring of dynamic AMPK activity within living cells with greatly enhanced sensitivity.

### Differential regulation of AMPK activity at the mitochondria and lysosome

**Fig. 2:**
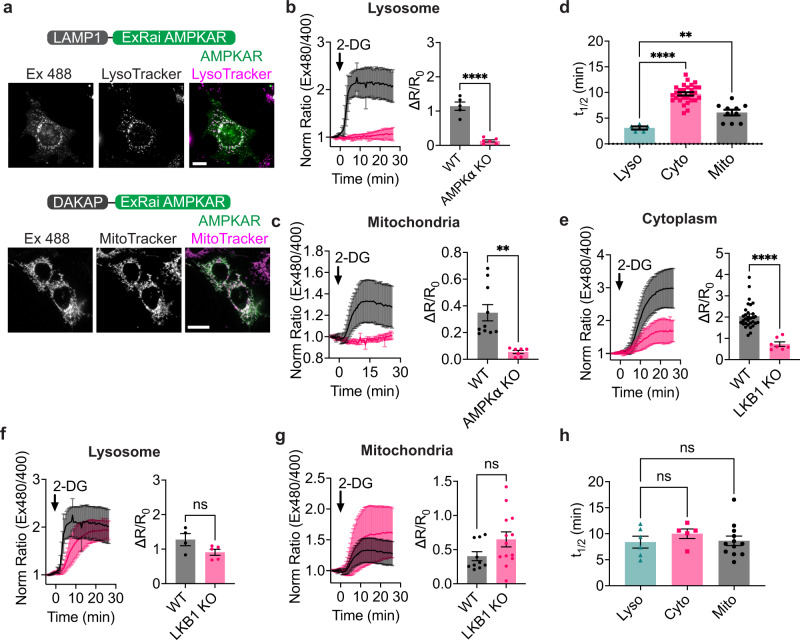
Lysosomal AMPK activity is rapidly induced in an LKB1-dependent manner. **a** (Top) Domain layout and representative image of lyso-ExRai AMPKAR in MEFs stained with the lysosomal marker LysoTracker Red. (Bottom) Domain layout and representative image of mito-ExRai AMPKAR in MEFs stained with the mitochondrial marker MitoTracker Red. **b** Average response of lyso-ExRai AMPKAR in WT (black, *n* = 5 cells from two experiments) and AMPKα KO MEFs (pink, *n* = 6 cells from two experiments) to 2-DG (40 mM) stimulation, along with maximum ratio change (*p* = 9.66 × 10^−6^, unpaired *t*-test, two-tailed). **c** Average response of mito-ExRai AMPKAR in WT (black, *n* = 10 cells from five experiments and AMPKα KO MEFs (pink, *n* = 7 cells from three experiments) treated with 2-DG (40 mM) along with maximum ratio change (**p = 0.0012, unpaired *t*-test, two-tailed). **d** Time to half-maximal response (*t*_1/2_) of lyso-ExRai AMPKAR (teal), cytoplasmic ExRai AMPKAR (pink), and mito-ExRai AMPKAR (black) following treatment with 2-DG (**p = 0.0033; ****p = 4.43 × 10^−10^, one-way ANOVA with Dunnett’s multiple comparisons test). **e** Average response to 2-DG (40 mM) stimulation of cytoplasmic ExRai AMPKAR in WT (black, reproduced from Fig. 1e) and LKB1 KO MEFs (pink, *n* = 7 cells from four experiments), along with maximum ratio changes (****p = 1.57 × 10^−6^, unpaired *t*-test, two-tailed). **f** Average response to 2-DG (40 mM) stimulation of lyso-ExRai AMPKAR in WT (black, reproduced from **b**) and LKB1 KO MEFs (pink, *n* = 5 cells from four experiments), along with maximum ratio changes (ns *p* = 0.084, unpaired *t*-test, two-tailed). **g** Average response to 2-DG (40 mM) stimulation of mito-ExRai AMPKAR in WT (black, reproduced from **c**) and LKB1 KO MEFs (pink, *n* = 13 cells from five experiments), along with maximum ratio changes (ns *p* = 0.09, unpaired *t*-test, two-tailed). **h** *t*_1/2_ of lyso-ExRai AMPKAR (teal, *n* = 6 cells from four experiments), cytoplasmic ExRai AMPKAR (pink, *n* = 5 cells from four experiments), and mito-ExRai AMPKAR (black, *n* = 10 cells from five experiments) following treatment with 2-DG in LKB1 KO MEFs (ns *p* ≥ 0.55, one-way ANOVA with Dunnett’s multiple comparisons test). For all figures, time courses show the mean ± SD, dot plots show the mean ± SEM. Scale bars, 20 µm.

The mitochondria and lysosome represent key signaling locations for AMPK^11,28,31–35^. Notably, results from western blotting assays have indicated that lysosomal pools of AMPK are preferentially activated under glucose deprivation, due to a lysosomal-localized AMPK regulatory complex^36^, while mitochondrial AMPK is activated under more severe nutrient stress^11^. However, dynamic AMPK activities have not be systematically analyzed at these two locations. Therefore, we deployed ExRai AMPKAR to better understand the spatiotemporal dynamics of AMPK at the lysosome and mitochondria. To specifically investigate lysosomal and mitochondrial AMPK activity, we fused ExRai AMPKAR to lysosome-associated membrane protein 1 (LAMP1) or a mitochondria-targeting sequence from dual specific A-kinase anchoring protein 1 (DAKAP1), respectively (Fig. 2a). We expressed the targeted reporters in either WT or AMPKα KO MEFs and confirmed targeted ExRai AMPKARs were well-localized to each respective compartment by performing fluorescence recovery after photobleaching (FRAP) on each reporter (Supplementary Fig. 2a). Comparison of basal starting intensity revealed ExRai AMPKAR localized to membranous organelles had a higher starting ratio than cytoplasmic ExRai AMPKAR, consistent with our previous findings^16^ (Supplementary Fig. 2b). WT and AMPKα KO MEFs expressing either lysosomal-targeted ExRai AMPKAR or mitochondrial-targeted ExRai AMPKAR were treated with multiple concentrations of 2-DG (Fig. 2b, c and Supplementary Fig. 2c). We compared the time-to-half-maximum (*t*_1/2_) of localized ExRai AMPKAR responses to 2-DG to identify kinetic differences amongst these locations. Lysosomal activity increased more rapidly than either cytoplasmic or mitochondrial activity (*t*_1/2_ = 3.09 ± 0.30 min, *p* ≤ 0.0033; Fig. 2d).

Next, we sought to identify the mechanism underlying rapid accumulation of AMPK activity at the lysosome. Lysosomal regulation of AMPK activity is thought to occur via a multi-protein complex which coordinates the lysosomal localization of AMPK and its upstream kinase LKB1^32,33^. We hypothesized that LKB1 plays a critical role in the fast kinetics of lysosomal AMPK activity compared to other locations. We therefore compared AMPK activity in WT and LKB1 knockout MEFs (LKB1 KO^37^, Supplementary Fig. 2d). As a control, LKB1 KO significantly reduced 2-DG-induced cytoplasmic AMPK activity, consistent with the established prominent role of LKB1 in mediating 2-DG-induced AMPK activity^38^ (2-DG ∆*R*/*R*_0_ WT MEF 2.04 ± 0.10; LKB1 KO MEF 0.73 ± 0.11, *p* < 0.0001; Fig. 2e). When we examined the 2-DG-induced lysosomal AMPK activity, we found that LKB1 KO did not affect the response amplitude of lyso-ExRai AMPKAR; strikingly the kinetics of 2-DG-induced lysosomal AMPK activity were substantially slower in LKB1 KO MEFs than in WT MEFs (*t*_1/2_ 8.39 ± 1.14 min, *p* = 0.0026; Fig. 2f). Unexpectedly, the absence of LKB1 had minimal effect on mitochondrial AMPK activity in response to 2-DG (Fig. 2g and Supplementary Fig. 2e). The kinetics of AMPK activity at the cytoplasm, mitochondria and lysosome were similar in LKB1 KO MEFs (Fig. 2h), indicating that LKB1 KO eliminated the kinetic advantage of lysosomal AMPK activity. These results suggest that the presence of LKB1 drives the fast kinetics of AMPK activity at the lysosome, most likely through close localization of LKB1 and AMPK at this organelle.

The lack of effect of LKB1 KO on the amplitude of lysosomal and mitochondrial AMPK activity prompted us to probe of the role of CaMKK2. 2-DG has been reported to increase intracellular calcium^19^, which could activate AMPK through CaMKK2. As only cytoplasmic AMPK activity was significantly diminished in the absence of LKB1, it is possible the substituting upstream kinases, such as CaMKK2, could allow for activity around membranes. To determine if CaMKK2 is involved in 2-DG-induced AMPK activity, we generated CaMKK2 KO MEFs using CRISPR/Cas9 (Supplementary Fig. 2f). We then measured AMPK activity in the cytoplasm, at the mitochondria, and lysosome (Supplementary Fig. 2g). We found that loss of CaMKK2 significantly decreased lysosomal AMPK activity (∆*R*/*R*_0_ 0.42 ± 0.11, *p* = 0.0013), suggesting CaMKK2 is important for maximal AMPK activity in response to 2-DG. In addition, loss of CaMKK2 significantly suppressed 2-DG-induced cytoplasmic and mitochondrial AMPK activity measured by ExRai AMPKAR (cytoplasm ∆*R*/*R*_0_ 1.06 ± 0.11, *p* < 0.0001; mitochondria ∆*R*/*R*_0_ 0.17 ± 0.0.041, *p* = 0.0015). Taken together, our findings reveal spatially distinct regulatory control of LKB1 over AMPK activity in response to 2-DG, whereas loss of CaMKK2 significantly suppresses AMPK activity throughout the cell.

### AMPK activity induced by allosteric activators exhibits a distinct spatial profile

Allosteric activation of AMPK, independent of energy stress or calcium signaling, is an attractive therapeutic route for treating diabetes and other metabolic disorders^39^. Toward this goal, several synthetic ligands which bind the ADAM site have been designed^40–42^, including MK-8722^43,44^. Recent work has also identified endogenous ligands for the ADAM site^45^. However, how ADAM site activators influence spatially compartmentalized AMPK signaling is not clear. Mechanistically, the importance of upstream kinases for ADAM site activation remains controversial^46,47^, because ADAM site activators protect AMPK from dephosphorylation by phosphatases^3,46,48^. As kinase and phosphatase activities can vary based on location^12,13^, we hypothesized there could be spatially distinct differences in allosteric activator induced AMPK activity. To test this hypothesis, we set out to use ExRai AMPKAR to profile spatiotemporal AMPK activity in response to allosteric activation via the ADAM site and determine the importance of the upstream kinase LKB1 for spatially defined, allosterically activated AMPK.

**Fig. 3:**
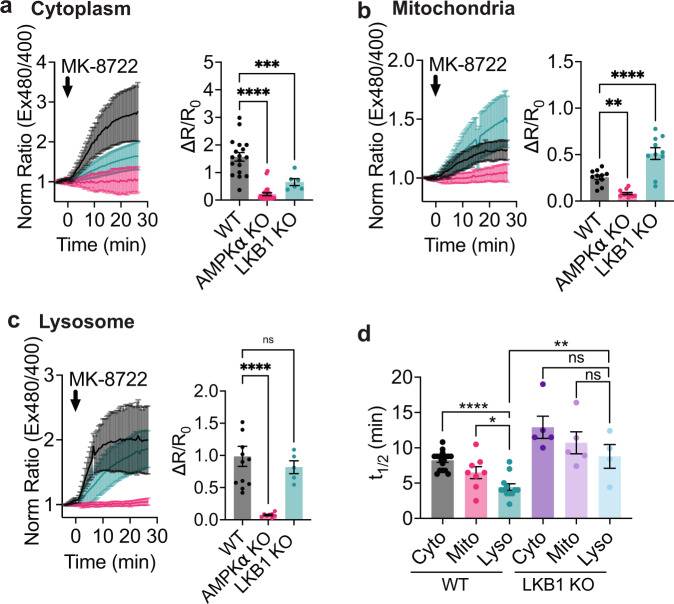
AMPK activity induced by MK-8722 exhibits distinct spatiotemporal dynamics. **a** Average response of ExRai AMPKAR in WT (black, *n* = 18 cells from three experiments), AMPKα KO (pink, *n* = 25 cells from three experiments), and LKB1 KO MEFs (teal, *n* = 6 cells from four experiments) treated with MK-8722 (500 nM) along with maximum ratio change (***p = 0.002, ****p < 1 × 10^−10^, one-way ANOVA with Dunnett’s multiple comparisons test). **b** Average response of mito-ExRai AMPKAR in WT (black, *n* = 11 cells from four experiments), AMPKα KO (pink, *n* = 11 cells from three experiments), and LKB1 KO MEFs (teal, *n* = 10 cells from five experiments) treated with MK-8722 (500 nM) along with maximum ratio change (**p = 0.0048, ****p = 9.76 × 10^−5^, one-way ANOVA with Dunnett’s multiple comparisons test). **c** Average response of lyso-ExRai AMPKAR in WT (black, *n* = 12 cells from three experiments), AMPKα KO (pink, *n* = 8 cells from three experiments) and LKB1 KO MEFs (teal, *n* = 5 cells from four experiments) to MK-8722 (500 nM) stimulation, along with maximum ratio change (ns = 0.66, ***p < 0.0001, one-way ANOVA with Dunnett’s multiple comparisons test). **d** *t*_1/2_ of cytoplasmic ExRai AMPKAR (black, *n* = 19 cells from three experiments), mito-ExRai AMPKAR (pink, *n* = 11 cells from four experiments), and lyso-ExRai AMPKAR (teal, *n* = 12 cells from three experiments) expressed in WT MEFs, and cytoplasmic ExRai AMPKAR (dark purple, *n* = 5 cells from four experiments), mito-ExRai AMPKAR (light purple, *n* = 5 cells from five experiments), and lyso-ExRai AMPKAR (light blue, *n* = 5 cells from four experiments) expressed in LKB1 KO MEFs following treatment with MK-8722 (*p = 0.0203; ****p = 1.47 × 10^−6^, ns ≥0.18, one-way ANOVA with Dunnett’s multiple comparisons test, **p = 0.0031, unpaired *t*-test, two-tailed). For all figures, time courses show the mean ± SD, dot plots show the mean ± SEM.

We first measured MK-8722-induced cytoplasmic AMPK activity in WT and AMPKα KO MEFs, finding MK-8722 strongly induced AMPK activity in the cytoplasm (Fig. 3a). We assessed the necessity of LKB1 for MK-8722-mediated AMPK activity. In LKB1 KO MEFs, MK-8722 induced cytoplasmic AMPK activity was significantly reduced compared with WT MEFs (WT: ∆*R*/*R*_0_ = 1.57 ± 0.16 vs. LKB1: ∆*R*/*R*_0_ = 0.66 ± 0.12, *p* < 1 × 10^−10^). These data suggest that LKB1 is required for the maximal cytoplasmic AMPK activity induced by the allosteric activator MK-8722.

Next, we examined mitochondrial and lysosomal AMPK activity induced by MK-8722. WT and AMPKα KO MEFs expressing mitochondrial- or lysosomal-targeted ExRai AMPKAR were treated with MK-8722 (Fig. 3b, c). MK-8722 robustly induced AMPK activity at these locations. We found lysosomal AMPK activity was more rapid than either mitochondrial or cytoplasmic AMPK activity (4.44 ± 0.47 min, *p* < 0.0001; Fig. 3d), suggesting that there are spatially distinct differences in AMPK activity induced by allosteric activators.

LKB1 KO also showed differential effect on MK-8722 induced subcellular AMPK activities. In LKB1 KO cells expressing lysosomal-targeted ExRai AMPKAR, we found MK-8722 still induced strong lysosomal AMPK activity, but with slower kinetics compared to WT MEFs, like that observed with 2-DG treatment (*t*_1/2_ 8.79 ± 1.68 min, *p* = 0.0031; Fig. 3c, d). On the other hand, the absence of LKB1 had minimal effect on mitochondrial AMPK activity in response to MK-8722 (Fig. 3b and Supplementary Fig. 2h), suggesting allosterically induced mitochondrial AMPK activity does not require an upstream kinase or another basally active upstream kinase is present at the mitochondria^19^. Together, we found the effect of ADAM site activation is not only dependent on AMPK and ADAM site ligands, but also relies on regulatory mechanisms, like upstream kinases, that are embedded in the spatiotemporal signaling network.

### Nuclear AMPK activity measured using ExRai AMPKAR

AMPK has been implicated in the regulation of various nuclear targets^8,9^, and AMPK subunits have been reported to directly localize to the nucleus^49^. However, how nuclear AMPK activity is induced remains controversial^9,10^. Biosensor-based studies have thus far been unable to conclusively shed light on the presence of nuclear activity^16,17,50^, and some studies suggest that AMPK is active in the nucleus in response to energy stress and others failed to detect any nuclear AMPK activity^11,51–54^. We therefore hypothesized that the enhanced sensitivity of ExRai AMPKAR would allow us to provide a more definitive view of nuclear AMPK activity.

**Fig. 4:**
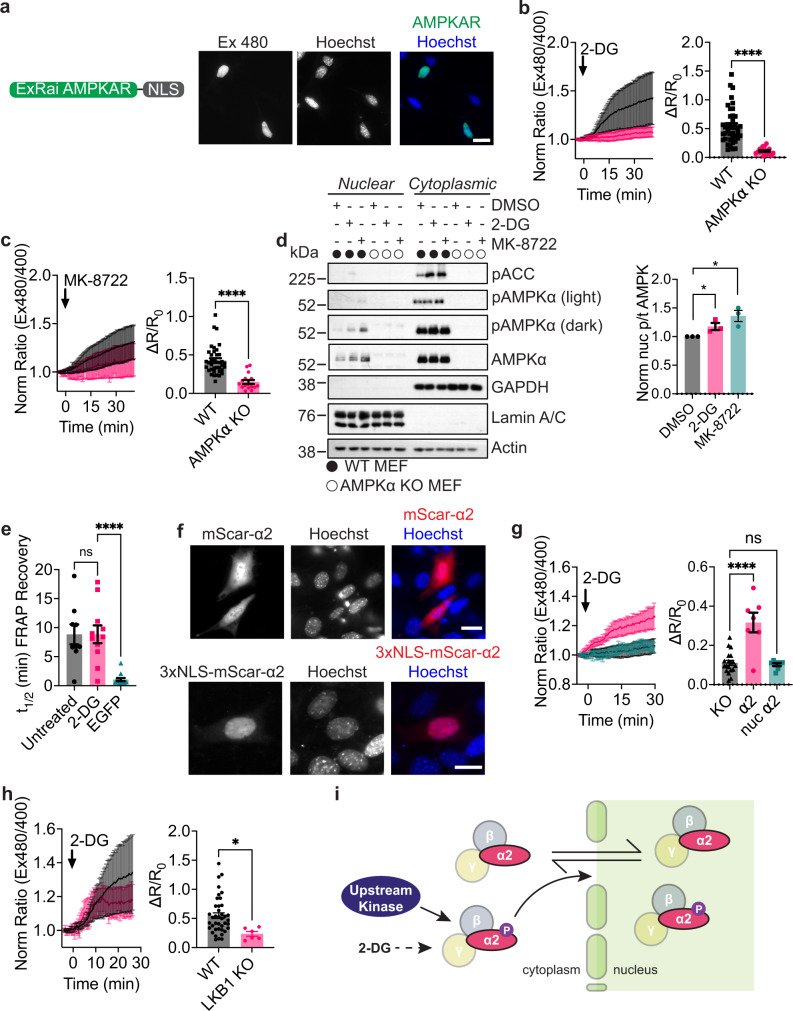
Nuclear AMPK activity measured using ExRai AMPKAR. **a** Domain layout and representative image of ExRai AMPKAR-NLS expressed in MEFs stained with Hoechst nuclear marker. **b** Average response of ExRai AMPKAR-NLS in either WT (black, *n* = 46 cells from five experiments) or AMPKα KO MEFs (pink, *n* = 19 cells from four experiments) treated with 2-DG (40 mM), along with maximum ratio change (****p = 1.51 × 10^−7^, unpaired *t*-test, two-tailed). **c** Average response of ExRai AMPKAR-NLS in either WT (black, *n* = 38 cells from nine experiments) or AMPKα KO MEFs (pink, *n* = 16 cells from two experiments) treated with MK-8722 (500 nM), along with maximum ratio change (****p = 6.35 × 10^−7^, unpaired *t*-test, two-tailed). **d** Western blot of nuclear-fractionated MEFs treated with DMSO, 2-DG (40 mM), or MK-8722 (500 nM) for 60 min. Quantification from three independent trials (*p = 0.047 DMSO vs. 2-DG; 0.020 DMSO vs. MK-8722, unpaired *t*-test, two-tailed). Full blots are shown in Source Data. **e** Half-time of FRAP recovery (min) for nuclear EGFP-AMPKα2 in AMPKα KO MEFs either without (*n* = 9 cells from three experiments) or with 2-DG stimulation (40 mM, *n* = 11 cells from three experiments) immediately before FRAP experiment began, or for EGFP alone (*n* = 13 cells from two experiments; ns *p* = 0.999; ****p < 0.0001, one-way ANOVA with Dunnett’s multiple comparisons test). **f** Representative images of mScarlet-AMPKα2 or 3xNLS-mScarlet-AMPKα2 in MEFs stained with Hoechst nuclear marker. **g** Average 2-DG (40 mM)-stimulated response of AMPKα KO MEFs expressing ExRai AMPKAR-NLS alone (black, reproduced from **b**) or co-expressing mScarlet-AMPKα2 (pink, *n* = 7 cells from two experiments) or 3xNLS-mScarlet-AMPKα2 (teal, *n* = 8 cells from four experiments), along with maximum ratio change (ns *p* = 0.96; ****p = 8.67 × 10^−7^, one-way ANOVA with Dunnett’s multiple comparisons test). **h** Average response of ExRai AMPKAR-NLS in either WT (black, reproduced from **b**) or LKB1 KO MEFs (pink, *n* = 6 cells from four experiments) treated with 2-DG (40 mM), along with maximum ratio change (*p = 0.021, unpaired *t*-test, two-tailed). **i** Mechanism of 2-DG-induced nuclear AMPK activity where nuclear AMPK activity in response to 2-DG is initiated in the cytoplasm dependent on upstream kinases, after which AMPK then translocates into the nucleus to phosphorylate nuclear targets. For all figures, time courses show the mean ± SD, dot plots show the mean ± SEM. Scale bars, 20 µm.

ExRai AMPKAR was fused to a nuclear localization sequence (NLS), and we confirmed ExRai AMPKAR nuclear localization by co-localization with nuclear markers and FRAP (Fig. 4a and Supplementary Fig. 3a). In WT MEFs, we observed significant AMPK activity in the nucleus following treatment with different concentrations of 2-DG, with minimal activity detected in AMPKα KO MEFs (WT: ∆*R*/*R*_0_ = 0.55 ± 0.05 vs. AMPKα KO: ∆*R*/*R*_0_ = 0.11 ± 0.013, *p* < 0.0001; Fig. 4b and Supplementary Fig. 3b). Similarly, MK-8722 induced nuclear AMPK activity in WT MEFs, whereas AMPKα KO MEFs showed minimal responses from nuclear localized ExRai AMPKAR (WT: ∆*R*/*R*_0_ = 0.43 ± 0.030 vs. AMPKα KO: ∆*R*/*R*_0_ = 0.15 ± 0.026, *p* < 0.0001; Fig. 4c). In parallel, we directly probed for phosphorylated AMPKα in the nuclei of WT MEFs using nuclear fractionation. Cells were treated with DMSO, 2-DG, or MK-8722, and nuclear fractions collected and probed for phosphorylated AMPKα via western blotting. We found that along with cytoplasmic targets of AMPK becoming phosphorylated following treatment with 2-DG or MK-8722, nuclear pools of AMPKα were phosphorylated as well (Fig. 4d). Thus, our results provide clear evidence of nuclear AMPK activity induced by both cellular stress and allosteric activation.

### Nuclear AMPK activity requires AMPKα2 nucleo-cytoplasmic shuttling

We next investigated the mechanism behind 2-DG-induced nuclear AMPK activity. We considered two possible scenarios for nuclear AMPK activity. The first scenario considers AMPK to reside within the nucleus and become activated in situ. In the second scenario, we considered AMPK to shuttle between the cytoplasm and nucleus, with AMPK activity being initiated in the cytoplasm, followed by translocation of active AMPK into the nucleus. To differentiate between these two possibilities, we first sought to determine if AMPK translocates into the nucleus under basal and stimulated conditions. For this purpose, we focused on AMPKα2, which has been detected in both the cytoplasm and nucleus^49^. To measure AMPK translocation, we performed FRAP experiments and monitored the nuclear intensity of EGFP-tagged AMPKα2 (EGFP-AMPKα2) in AMPKα KO MEFs after photobleaching of the nuclei. Under basal conditions, nuclear EGFP-AMPKα2 intensity was fully recovered within 30 min (*t*_1/2_ = 8.84 ± 1.72 min; Fig. 4e and Supplementary Fig. 3c). EGFP-AMPKα2 nuclear intensity showed similar recovery kinetics following treatment with 2-DG (*t*_1/2_ = 8.86 ± 1.53 min; *p* = 0.999). Control cells expressing EGFP alone showed full recovery of nuclear intensity within only a few minutes (*t*_1/2_ = 1.06 ± 0.26 min, *p* < 0.0001), indicating that the slower recovery time for nuclear EGFP-AMPKα2 fluorescence intensity is due to AMPKα2 itself and not the EGFP tag.

We then compared the *t*_1/2_ of EGFP-AMPKα2 import into the nucleus to that of 2-DG-induced nuclear AMPK activity. 2-DG induced nuclear AMPK activity is slower than the half-time for EGFP-AMPKα2 translocation into the nucleus (*t*_1/2_ 15.53 ± 1.14 min, Supplementary Fig. 3d). This supports our second scenario; whereby activated AMPK can translocate from the cytoplasm to the nucleus to induce nuclear signaling. To test if nuclear AMPK activity is dependent on AMPK shuttling into the nucleus, we sought to localize AMPKα to the nucleus and measure nuclear AMPK activity in AMPKα KO MEFs. To sequester AMPK in the nucleus, we considered known nuclear localization and exclusion sequences present in AMPKα2^55,56^. However, mutating either the nuclear exclusion or localization sequence on AMPKα2 yielded no significant effect on the nuclear localization of EGFP-tagged AMPKα2 in HEK293T cells (Supplementary Fig. 3e). Indeed, the effects of these sequences on nuclear AMPK activity are thought to be cell-type specific. For example, the putative nuclear localization signal was identified in mouse myoblasts and found to be leptin-dependent over the course of several hours, and not functional in HEK293T cells^55,56^. Therefore, we set out to further understand how nuclear AMPK activity is regulated by using a subcellular targeting approach.

We generated a nuclear-targeted AMPKα2, 3xNLS-mScarlet-AMPKα2, with untargeted mScarlet-AMPKα2 as a control. While mScarlet-AMPKα2 can translocate from the cytoplasm to the nucleus, the addition of three tandem NLS efficiently localizes mScarlet-AMPKα2 to the nucleus and minimizes its exposure to the cytoplasm (Fig. 4f). We next examined the ability of these AMPKα2 constructs to rescue nuclear AMPK activity. Untargeted mScarlet-AMPKα2 restored nuclear AMPK activity following treatment with 2-DG in AMPKα KO MEFs (∆*R*/*R*_0_ = 0.39 ± 0.063, *p* < 0.0001; Fig. 4g), indicating that AMPKα2 is adequate for nuclear AMPK activity. However, expression of 3xNLS-mScarlet-AMPKα2 was insufficient to restore nuclear AMPK activity in AMPKα KO MEFs following treatment with 2-DG (∆*R*/*R*_0_ = 0.10 ± 0.0075, *p* = 0.96). These results indicate that AMPK sequestered in the nucleus cannot respond to 2-DG stimulation and further support a mechanistic model of translocation-based nuclear AMPK activity. This suggests that nuclear AMPK activity will show a similar dependence on upstream kinases as cytoplasmic AMPK activity. Indeed, in LKB1 KO MEFs expressing nuclear ExRai AMPKAR, we found that nuclear AMPK activity was suppressed following 2-DG treatment (∆*R*/*R*_0_ = 0.24 ± 0.04, *p* = 0.021; Fig. 4h). We validated these results in HeLa cells with and without LKB1 expression (Supplementary Fig. 3f). In CaMKK2 KO MEFs, we saw similar suppression of nuclear AMPK activity following 2-DG treatment (Supplementary Fig. 3g). From our work, we propose a mechanistic model in which nuclear AMPK activity in response to 2-DG is initiated in the cytoplasm dependent on upstream kinases, after which AMPK then translocates into the nucleus to phosphorylate nuclear targets (Fig. 4i).

## Discussion

Genetically encoded fluorescent protein-based kinase activity reporters have become essential tools to investigate compartmentalized signaling networks. In the present study, we designed and used a single-fluorophore AMPK activity reporter, ExRai AMPKAR, the most sensitive AMPK activity reporter thus far developed. While FRET-based AMPKARs have been successfully used to interrogate AMPK activity^16–19^, the limited dynamic range of FRET-based reporters significantly limits their application. ExRai AMPKAR has over 3-times higher dynamic range, enabling detection of subtle changes in AMPK activity. This allowed us to clearly detect nuclear AMPK activity, which has posed a challenge for FRET-based AMPKARs^16,17^. Future engineering efforts will focus on enhancing the dynamic range of some of the subcellularly targeted variants, as mito-ExRai AMPKAR exhibited a reduced dynamic range compared to an untargeted counterpart, presumably due to close proximity with membranes, as we have seen previously with other targeted biosensors^16,57^. We found that switching the reporting unit could affect the kinetics of the response (Fig. 1f)^20^, but the kinetics of ExRai AMPKAR correlate well with the phosphorylation of endogenous AMPK substrates (Supplementary Fig. 1c–e). Like FRET AMPKAR^16,19^, ExRai AMPKAR could be used to assess basal AMPK activities, especially by observing the dynamic changes induced by inhibitor treatment at the resting state, a strategy which has been successfully used with FRET-based kinase activity reporters^14,58–60^. Overall, ExRai AMPKAR represents a significant advancement in the sensitive detection of subcellular AMPK activity, and enables the robust visualization of subtle, subcellular AMPK signaling events.

Using ExRai AMPKAR, we interrogated the spatiotemporal dynamics of AMPK activity in response to two stimuli: cellular stress and allosteric activation by ADAM site ligands. We found that lysosomal AMPK activity was more rapidly induced compared to mitochondrial or cytoplasmic AMPK activity by either cellular stress or allosteric activation. As the lysosome has been suggested to function as a signaling hub^36,39^, the rapid accumulation of AMPK activity at the lysosome could provide an advantage to substrates in proximity. Interestingly, we show that irrespective of the mode of activation, via cellular stress or allosteric activation, the kinetic trend for spatial AMPK activity is the same, suggesting the lysosome is a privileged location for AMPK to sense and respond to a variety of different activating signals.

Building on these findings, we investigated the necessity of LKB1 for subcellular AMPK activity. At the lysosome, loss of LKB1 was associated with slower kinetics of AMPK activity, consistent with the demonstration of an AMPK regulatory complex at the lysosome consisting of AXIN, LKB1, and lysosomal membrane proteins such as vATPase and Ragulator^33^. While cytoplasmic AMPK activity was significantly diminished in the absence of LKB1, we found that LKB1 is not required for maximal lysosomal or mitochondrial AMPK activity. Consistent with our findings, AMPK activity has been detected in LKB1-deficient cells stimulated with 2-DG, which was abolished upon deletion or inhibition of CaMKK2^19^. In our studies, we found CaMKK2 is needed for maximal AMPK activity in response to 2-DG, suggesting both LKB1 and CaMKK2 are needed for rapid and maximal AMPK activity in response to cellular stress. The necessity of upstream kinases for the effect of ADAM site activators is debated^46,47^, and our studies show location-specific dependence of MK-8722 on LKB1 to stimulate AMPK activity. While MK-8722 did not maximally induce AMPK activity in the cytoplasm in the absence of LKB1, impacts on MK-8722-induced AMPK activity at the mitochondria and lysosome were minimal. While our studies investigate the spatiotemporal roles of upstream kinases of AMPK, spatial differences in AMPK activity could also be due to subcellular differences in phosphatase activities^61–63^, requiring further investigation.

With the increased sensitivity of ExRai AMPKAR, we report nuclear AMPK activity in response to treatment with 2-DG and MK-8722 and propose a mechanism for 2-DG-induced nuclear AMPK activity (Fig. 4i). Basally, AMPKα2 can translocate between the cytoplasm and nucleus. As macromolecules larger than 40 kDa typically require the assistance of nuclear transport factors to cross the nuclear membrane^64^, future studies are needed to identify the molecular determinants responsible for shuttling AMPK, which comprises three subunits with a combined molecular weight > 100 kDa^65^. Our FRAP experiments suggested that the kinetics of AMPK nuclear shuttling are not affected by 2-DG. However, subcellular fractionation revealed slight increases in nuclear AMPK levels upon 2-DG stimulation (Fig. 4d). Substantial translocation and accumulation of AMPKα subunits to the nucleus were reported after long-term treatment with leptin or adiponectin^56^, suggesting time- and stimulation-dependent accumulation of nuclear AMPK. Finally, we found that nuclear AMPK activity is reliant on LKB1 and CaMKK2. Others have reported that LKB1 is primarily active in the cytoplasm^66–68^, consistent with our model of LKB1 phosphorylating cytoplasmic AMPK, followed by nuclear translocation of phosphorylated AMPK to lead to subsequent nuclear activity. CaMKK2 is reported to be primarily localized to cytoplasm^69^, further suggesting nuclear AMPK activity originates in the cytoplasm. As our model suggests constant shuttling of AMPK between the cytoplasm and nucleus, it is possible that active AMPK is shuttled back to the cytoplasm, where it could be dephosphorylated. Taken together, our results present a model of nuclear AMPK activity.

Our nuclear studies were limited to investigation of AMPKα2-containing complexes. We focused on AMPKα2 due to the known nuclear localization of this subunit^49^. However, emerging evidence suggests that AMPKα1 is vital for calcium-induced nuclear AMPK activity. Etoposide treatment specifically induced CaMKK2-dependent AMPK activity in the nucleus, but only with AMPKα1-containing complexes, even in the presence of AMPKα2 complexes^70^. CaMKK2-dependent AMPK activity has also been reported in response to a variety of physiological and pharmacological agents which increase intracellular calcium^71–76^, also dependent on AMPKα1^74,76–78^. In this work, we have shown that AMPKα2 is sufficient for stress-induced nuclear AMPK activity. Input-dependent AMPKα isoform specificity could provide another level of control over nuclear AMPK activity. These additional regulatory controls would ensure precise tuning of nuclear AMPK activity and allow the kinase to discriminate between downstream effectors.

In summary, we have generated a single-fluorophore excitation-ratiometric AMPK activity reporter, which we have used to uncover mechanisms of compartmentalized AMPK activity. Using our reporter, precise and sensitive investigation into spatial AMPK signaling is now possible, which should lead to a better understanding of metabolic regulation throughout the cell.

## Methods

### Materials

2-deoxyglucose (2-DG, Sigma, D6134-250MG) was dissolved in 1x DPBS. SBI-0206965 (Cayman Chemical, 18477) and MK-8722 (Aobious, AOB33226) were dissolved in DMSO (Sigma-Aldrich). Hoescht 33342 (Cell Signaling, 4082S) and puromycin (Sigma-Aldrich, P9620) were dissolved in deionized water. For western blotting and immunostaining, antibodies from Cell Signaling Technologies (Denvers, MA USA) were used at 1:1000 dilution unless otherwise noted: P-ACC S79 (3661), P-AMPK T172 (2535), ACC (3662), AMPKα (2532), GAPDH (5174, 1:10,000), Laminin A/C (4777), LKB1 (3047), β-tubulin (2146, 1:10,000). From Sigma-Aldrich, anti-Actin (A5441) was diluted 1:10,000 and anti-Tubulin (T5168) was diluted 1:5000. From Santa Cruz, CaMKK2 (sc-100364) was diluted 1:500–1:1000. From Pierce, horseradish peroxidase-labeled goat anti-rabbit (PI31460) or anti-mouse (PI31430) was diluted 1:1000–1:10,000. Lipofectamine 2000 (11668019) was purchased from Thermo Fisher, and FuGENE HD (E2311) was purchased from Promega. MitoTracker Red (M22425) and LysoTracker Red (L7528) were purchased from Thermo Fisher and diluted in DMSO.

### Plasmids

All primers used for molecular cloning can be found in Supplementary Table 1. To generate ExRai AMPKAR, DNA fragments encoding cpGFP and FHA1 binding domain and linker pair candidates obtained for our protein kinase A sensor ExRai AKAR^21^ were digested with SacI and EcoRI restriction enzymes (Thermo Fisher, FD1134 and FD0275, respectively) and ligated into a SacI/EcoRI-digested pRSET-B backbone containing an AMPK substrate sequence. These constructs were then subcloned into the pcDNA3 vector via digestion with BamHI (Thermo Fisher FD1464) and EcoRI restriction enzymes. ExRai AMPKAR T/A was generated via Gibson Assembly using NEBuilder HiFi DNA Assembly Kit (New England Biolabs E2621) using primers 1–2. Mitochondrial (MAIQLRSLFPLALPGMLALLGWWWFFSRKKADP), and nuclear (PKKKRKVEDA) ExRai AMPKAR were made by PCR-amplifying ExRai AMPKAR using primers 3–6 followed by insertion into BamHI/EcoRI-digested vector backbones containing the indicated localization sequences. Lysosomal ExRai AMPKAR was made by inserting PCR-amplified lysosome-associated membrane protein 1 (LAMP1) generated using primers 7–8 into HindIII (Thermo Fisher FD0504)/BamHI-digested ExRai AMPKAR backbone. mScarlet-AMPKα2 and 3xNLS-mScarlet-AMPKα2 (NLS targeting: PKKKRKVDPKKKRKVDPKKKRKV) in pEGFP-N1 expression vector were generated via Gibson Assembly using primers 9–12. AMPKα2 mutants were generated using primers 13–14. Generation of gRNA-containing plasmids for knockout of CaMKK2 are described below. Successful clone generation was confirmed by Sanger sequencing (Genewiz). FRET-based ABKAR^18^ and mCherry-LKB1^18^ were previously reported. EGFP-AMPKα2^55^ was a gift from Jay Brenman (Addgene plasmid 30310). H2B-mCherry was a gift from Michael Davidson (Addgene plasmid 55055). pVSVg was a gift from Bob Weinberg (Addgene plasmid 8454). psPAX2 was a gift from Didier Trono (Addgene plasmid 12260). lentiCRISPR v2 was a gift from Feng Zhang (Addgene plasmid 52961).

### Cell culture and transfection

HEK293T cells were acquired from ATCC and cultured in Dulbecco’s modified Eagle medium (DMEM; Gibco 11885-084) containing 1 g/l glucose, 10% fetal bovine serum (FBS, Gibco 26140-079), and 1% (v/v) penicillin-streptomycin (Pen/Strep, Gibco 15140-122). WT MEFs, AMPK KO MEFs, and LKB1 KO MEFs were described previously^28,37^ and cultured in DMEM (Gibco 11995-065) containing 4.5 g/l glucose, pyruvate, and L-glutamine, supplemented with 10% FBS and 1% Pen-Strep. CaMKK2 KO MEFs were generated as described below and were cultured in the same MEF medium. Cos7 cells were obtained from ATCC and cultured under the same conditions as MEFs. All cells were grown in humidified incubators kept at 37 °C and 5% CO_2_ (HeraCell) and checked for mycoplasma using Hoechst staining. For transfection, cells were plated on 35-mm glass-bottomed dishes (CellVis D35-14-1.5-N). Cells were transfected 2–24 h after plating. HEK293T cells and Cos7 cells were transfected with DNA using Lipofectamine 2000, and MEFs were transfected with DNA using FuGENE HD. Cells were imaged 24–48 h after transfection.

### Generation of CaMKK2 KO MEFs using CRISPR/Cas9

CAMKK2 knockouts were generated using the LentiCRISPR v2 strategy as previously described^79,80^. Guide RNAs were designed using Benchling guide design tool (www.benchling.com/crispr, N- GCCAGCTTGACAACACCAT -C). Oligonucleotides from IDT were phosphorylated, annealed, and ligated into LentiCRISPR vector v2-Puro digested with BsmB1 (New England Biolabs R0739). Lentiviruses were produced by co-transfection of the lentiviral backbone constructs and packaging plasmids pVSVg and psPAX2 in 293T cells. Lipofectamine 2000 was used as a transfection reagent at a ratio of 3:1 lipofectamine/DNA. Lentivirus was collected 72 h after transfection. MEFs were infected with 0.45µM-filtered lentiviral supernatant supplemented with polybrene (Sigma-Aldrich 107689) for 24 h. Cells recovered for 24 h and then were selected with 2.5 µl/ml puromycin. Polyclonal pools were screened for target deletion by western blot. The pools confirmed to stably express sgRNAs were single-cell sorted into 96 well plates. Monoclonal lines were expanded from single cells and their knockout status was then determined by western blot to use for subsequent experiments.

### Fluorescence imaging and image analysis

For all live cell imaging experiments, cells were washed and incubated in Hanks balanced salt solution (HBSS, Gibco 14065-056; buffered with 20 mM HEPES, pH 7.4 and supplemented with 2 g/l glucose) for at least 30 min at 37 °C prior to imaging. All imaging was performed in the dark at 37 °C. 2-DG (40 mM), MK-8722 (500 nM), and SBI-0206965 (30 µM) were added at the indicated times.

Time-lapse epifluorescence images were acquired on either a Zeiss AxioObserver Z1 microscope (Carl Zeiss) equipped with a plan-apochromat ×20/0.8 N/A and ×40/1.4 N/A objectives (Carl Zeiss) and CMOS Orca Flash 4.0 camera (Hamamatsu) enclosed in a custom incubator, or a Zeiss AxioObserver Z7 microscope equipped with a ×40/1.4 N/A objective, Prime 95B sCMOS camera (Photometrics), and stage-top incubator (Carl Zeiss). Imaging experiments were done using a modified version of the open-source MATLAB (Mathworks) and µmanager (Micro-Manager)-based MATScope imaging suite (GitHub, see Code Availability statement). Dual GFP excitation-ratio imaging was accomplished using ET405/40x and ET480/30x excitation filters with a T505dcxr dichroic, and a ET535/50 m emission filter. EGFP was imaged using an ET480/30x excitation filter with a T505dcxr dichroic, and ET535/50 m emission filter. mCherry and mScarlet were imaged using an HQ568/55x excitation filter with a Q600LPxr dichroic and HQ653/95 m emission filter. Dual cyan/yellow emission ratio imaging was performed using an ET420/20x excitation filter, a T4551pxt dichroic, and two emission filters (ET470/40 m for CFP and ET535/25 m for YFP). CFP imaging was done using ET420/20x excitation filter with a T4551pxt dichroic and AT470/40 m emission filter. YFP imaging was done using ET495/10x excitation filter with a T5151p dichroic and ET535/25 m emission filter. All filter sets were controlled by an external filter-exchanger (Prior Scientific or Ludl Electronic Products, Ltd). Exposure times ranged between 50 to 100 ms, and images were acquired every 15–60 s.

Image analysis for time-lapse imaging was done using custom MATLAB code. Regions of interest (ROI) were randomly selected in cells throughout the field of view. For localized biosensors, ROIs were selected around the mitochondria, a region of the cell containing lysosomes, or the entire nucleus. Raw fluorescence intensities were corrected for background fluorescence using a cell-free area. cpGFP excitation ratios (Ex480/400) and yellow/cyan FRET emission ratios were calculated for each time point. Ratios were normalized to values before drug stimulation. Maximum ratio changes (∆*R*/*R*_0_) were calculated as (*R*_max_ − *R*_0_)/*R*_0_, where *R* is the excitation or emission ratio. SNR for each cell was calculated by dividing the maximum ratio change by the standard deviation of the baseline before drug addition. Z-factors were calculated as 1 – (3*σ*_max response average_ + 3*σ*_baseline average_)/|*σ*_max response average_ − *σ*_baseline average_|. Further data analysis was done using Microsoft Excel Version 16. Graphs were plotted using GraphPad Prism 8 and 9 (GraphPad Software).

FRAP experiments were performed on a Nikon Ti2 C2 confocal microscope equipped with a 40×1.3 NA oil objective (Nikon), 405 nm, 488 nm, and 561 nm laser lines, C2-DUVB GaAsP detector, and Okolabs stage-top incubator. The microscope was controlled using NIS-Elements software (High content analysis package, Nikon). Nuclei were identified by H2B-mCherry expression. The entire nucleus was selected for photobleaching, or for biosensor, region of the cell expressing the biosensor. The ROI was photobleached for 1 s with 100% 488 nm laser power or 2 s with 100% 488 nm and 25% 405 nm laser power and observed for 3–25 min post-bleaching. Unnormalized intensity data was collected using NIS-Elements and normalized using Excel (Microsoft). FRAP curves were fit to a single exponential recovery equation using the curve fitting tool in FIJI Is Just ImageJ (FIJI)^81^, *y* = *a*(1 − *e*^*-bx*^) *+* *c*.

Pseudocolor images were created using FIJI.

### Western blotting

MEFs were plated in either 6- or 10-cm dishes in growth medium. Cells were treated with small molecules and scraped on ice and pelleted in PBS. PBS was aspirated and the cells where lysed in lysis buffer (3x radioimmunoprecipation assay buffer, protease inhibitor cocktail (Roche 1169749801), 1 mM phenylmethylsulfonyl fluoride, 1 mM sodium pervanadate, 1 mM sodium fluoride, and 25 mM calyculin A). Protein samples were separated using 4–15% SDS-PAGE (BioRad 456-1085) and transferred to nitrocellulose membranes. Membranes were blocked using 1% bovine serum albumin (Sigma-Aldrich 03116956001 in 1x TBS, 0.05% Tween-20) and incubated with primary antibodies overnight at 4 °C. After incubation with the appropriate horseradish peroxidase-conjugated secondary antibody, bands were visualized by chemiluminescence. Uncropped full blots are available in the Source Data.

### Nuclear fractionation

MEFs were seeded at a density of 6 × 10^6^ cells per 15-cm dish in growth medium 18 h prior to treatment. Small molecules were applied to cells and incubated for 60 min at 37 °C: DMSO (3 µl/ml), 2-DG (40 mM), MK-8722 (500 nM). Cells were scraped on ice and pelleted in PBS. PBS was aspirated and cells were taken forward for nuclear/cytoplasmic fractionation using NE-PER kit (Thermo Fisher Scientific, 78835) following manufacturer’s protocol, or for whole-cell lysate control samples. Fractionation was performed using WT MEFs and AMPK KO MEFs. Parallel whole-cell lysates were generated using CST lysis buffer (20 mM Tris, pH 7.5, 150 mM NaCl, 1 mM EDTA, 1 mM EGTA, 1% Triton X-100, 2.5 mM pyrophosphate, 50 mM NaF, 5 mM β-glycero-phosphate, 50 nM calyculin A, 1 mM Na_3_VO_4_, and protease inhibitors). Lysates were incubated at 4 °C for 15 min and cleared at 16,000 × *g* for 10 min at 4 °C. Total protein was normalized using the BCA Protein Assay kit (Pierce 23225).

### Statistics and reproducibility

Figure preparation and statistical analysis were performed using GraphPad Prism 8 or 9. For comparison of two parametric data sets, Student’s *t* test was used. Nonparametric tests were done using Mann–Whitney *U*. For comparing three or more sets of data, ordinary one-way ANOVA followed by multiple comparisons test was done. Statistical significance was defined as *p* < 0.05 with a 95% confidence interval. The number of cells analyzed (*n* cell) and number of independent experiments are reported in all figure legends. All time courses shown are the mean of all cells ± standard deviation unless otherwise noted. All dot plots shown depict the mean ± standard error of the mean.

### Reporting summary

Further information on research design is available in the Nature Research Reporting Summary linked to this article.

## Supplementary information

Supplementary Information

Reporting Summary

## Data Availability

The plasmids utilized in this article will be submitted to the non-profit plasmid repository, Addgene, for scientific sharing. Source data are provided with this paper.

## Source data

Source Data

## Code availability

Custom MATLAB code used for image acquisition and analysis is available at https://github.com/jinzhanglab-ucsd/MatScopeSuite (10.5281/zenodo.5908008).
