# Supplementary material for: Spatial regulation of AMPK signaling revealed by a sensitive kinase activity reporter
Source: Nat Commun. 2022 Jul 5;13:3856. doi: 10.1038/s41467-022-31190-x (PMC9256702; doi:10.1038/s41467-022-31190-x)
Supplement: Supplementary file 1 — Supplementary Information [file 41467_2022_31190_MOESM1_ESM.pdf]

Supplementary Figures and Tables

Supplementary Figure 1. Development and characterization of ExRai AMPKAR.

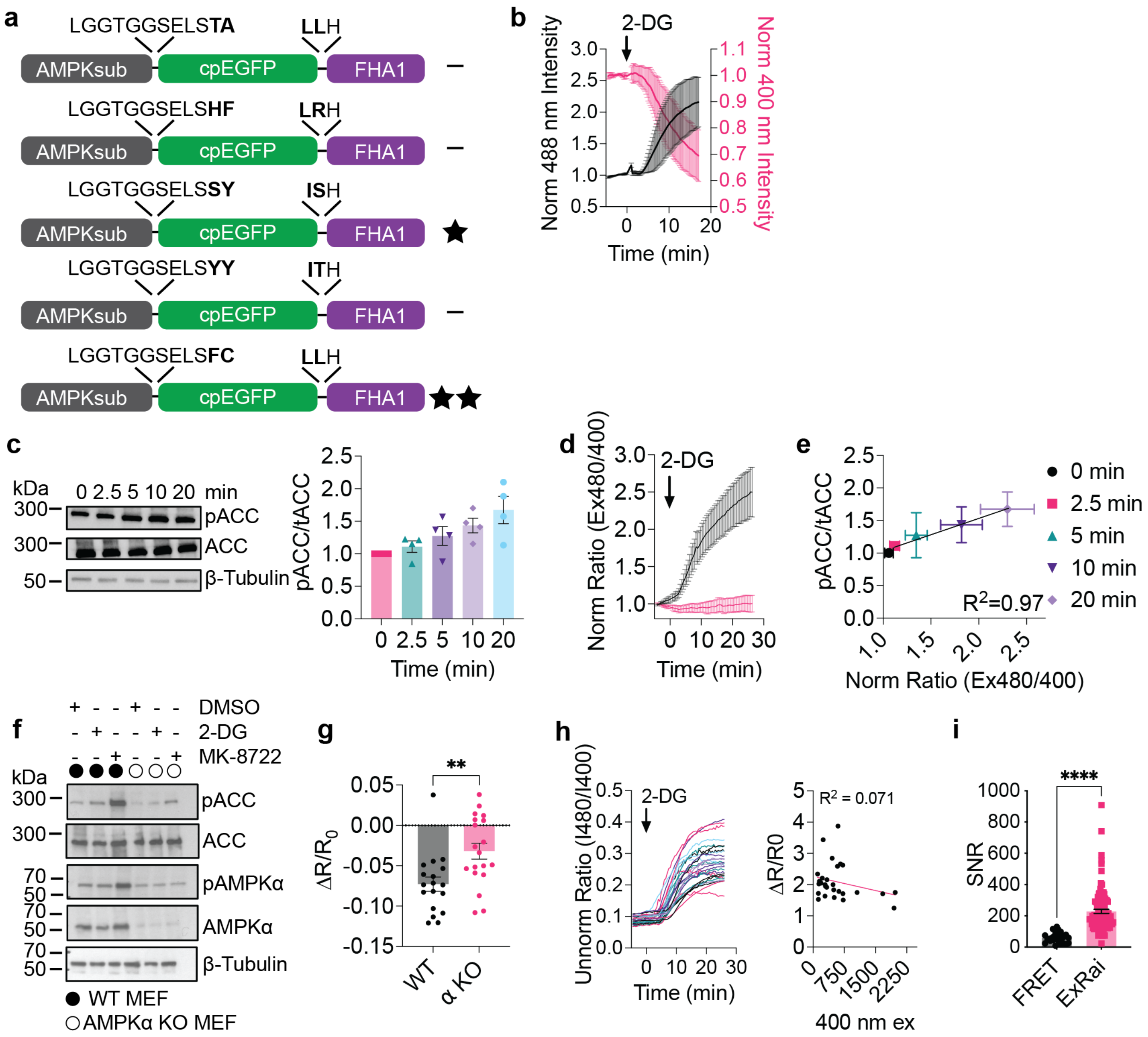

a, Domain structures of ExRai AMPKAR linker variants tested. Variants are scored based on performance: -, <40%; ★, 40-120%; ★★, >120% change in excitation ratio.

b, Average normalized 480 nm-excited fluorescence intensity (black), 400 nm-excited fluorescence intensity (pink) of Cos7 cells expressing ExRai AMPKAR in Cos7 cells treated with 2-DG (40 mM, n = 6 cells from 2 experiments).

c, Representative western blot and quantification of acetyl Co-A carboxylase (ACC) phosphorylation, an endogenous substrate of AMPK, in WT MEFs treated with 10 mM 2-DG for 0-20 min. Uncropped western blot shown in **Source Data**. Representative of 4 independent trials.

d, AMPK activity measured using ExRai AMPKAR following treatment with 10 mM 2-DG (WT MEFs black, n = 6 cells from 2 experiments; AMPKα KO MEFs pink, n = 6 cells from 3 experiments),

e, Correlation of ACC phosphorylation (reproduced from **Supplementary Fig 1c**) with ExRai AMPKAR response (reproduced from **Supplementary Fig 1d**) at indicated times. Data fit with linear regression and R^2 displayed on graph.

f, Western blot of AMPK activity in WT and AMPK KO MEFs treated with DMSO, 2-DG (40 mM), or allosteric AMPK activator MK-8722 (500 nM) for 30 min. Western blots are representative of at least 3 replicates. Full blot is shown in **Source Data**.

g, Ratio change ($\Delta R/R_0$) of ExRai AMPKAR expressed in WT (black, n = 18 cells from 4 experiments) or AMPK KO MEFs treated with SBI-0206965 (SBI, 30 μM; pink, n = 19 cells from 5 experiments, ** P = 0.0041, unpaired t-test, two-tailed).

h, (left) Unnormalized ExRai AMPKAR response in WT MEFs from **Fig 1e**. (right) ExRai AMPKAR response ($\Delta R/R_0$) from **Fig 1e**, plotted against the initial fluorescence with 400 nm excitation (Ex 400). Data fit using linear regression, R^2 displayed on graph.

i, Signal-to-noise ratio (SNR) of FRET-based AMPK reporter ABKAR (black, n = 25 cells from 2 experiments) and ExRai AMPKAR (pink, n = 86 cells from 5 experiments, ****P = 3.99×10^{-9}, unpaired t-test, two-tailed).

Time courses show the mean ± SD, dot plots are shown as mean ± SEM.

Supplementary Figure 2. Investigation of dynamics of cytoplasmic, mitochondrial, and lysosomal AMPK activity using ExRai AMPKAR.

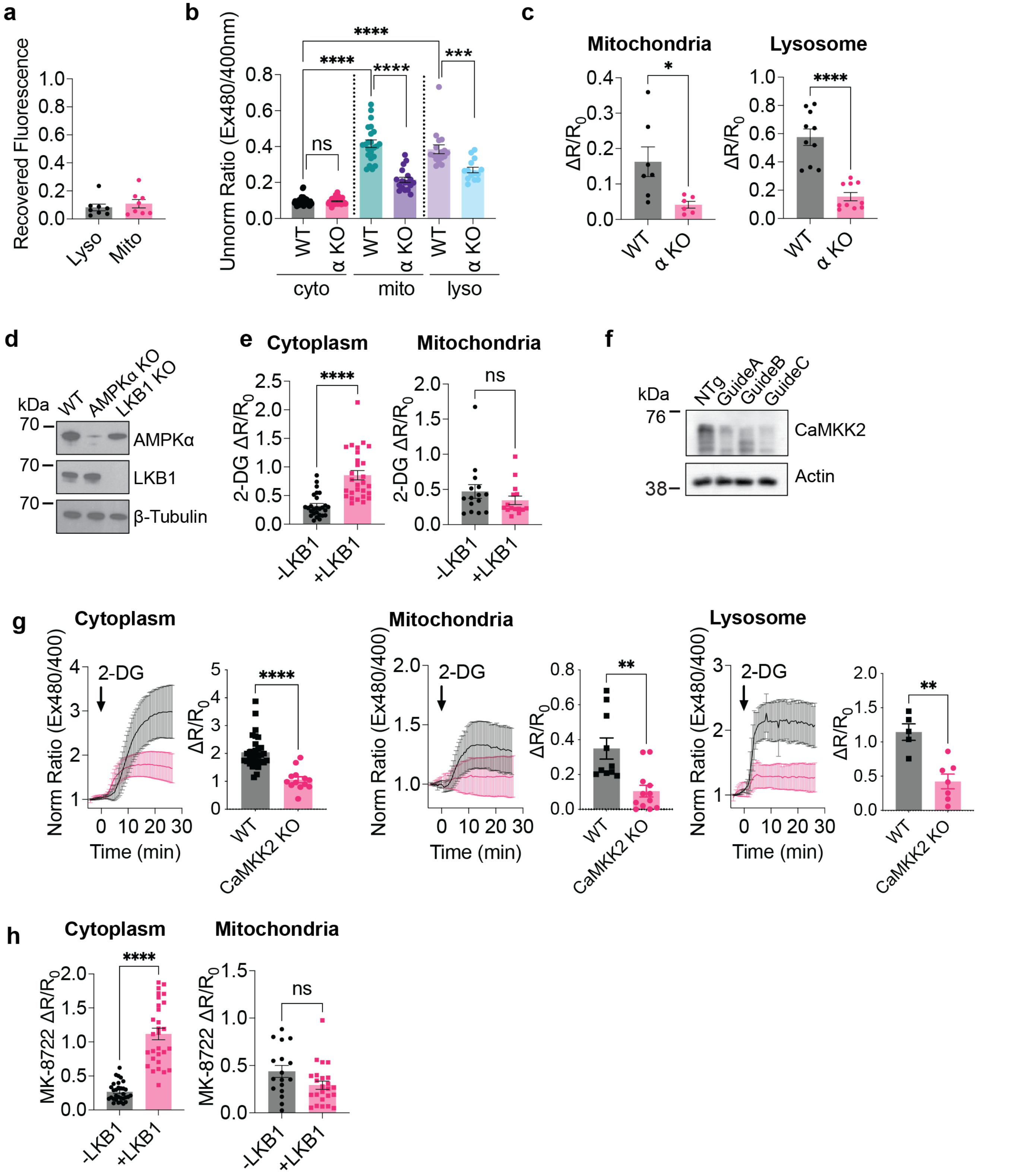

a, Percent fluorescence recovery after photobleaching (FRAP) of ExRai AMPKAR fused to targeting sequences for the lysosome (lyso, black, n = 8 cells from 2 experiments) or mitochondria (mito, pink, n = 8 cells from 2 experiments).

b, Unnormalized starting ratio of ExRai AMPKAR in the cytoplasm (WT MEFs black, n = 47 cells from at least 3 experiments; AMPKα KO MEFs pink, n = 40 cells from at least 3 experiments; P = 0.99, unpaired t-test, two-tailed), mitochondria (WT MEFs teal, n = 23 cells from at least 3 experiments; AMPK KO MEFs dark purple, n = 18 cells from at least 3 experiments; ****P = 4.39×10^{-9}, unpaired t-test, two-tailed, or lysosome (WT MEFs light purple, n = 17 from at least 3 experiments; AMPKα KO MEFs light blue n = 14 cells from at least 3 experiments; ***P = 0.0007, unpaired t-test, two-tailed). **** P<1x10-^{10}, ANOVA with Dunnett's multiple comparison's test.

c, AMPK activity following treatment with 10 mM 2-DG for 30 min at mitochondria (WT MEFs black, n = 7 cells from 3 experiments; AMPKα KO MEFs pink, n = 6 cells from 3 experiments; *P = 0.024, unpaired t-test, two-tailed) and lysosome (WT MEFs black, n = 10 cells from 5 experiments; AMPKα KO MEFs pink, n = 10 cells from 5 experiments; ****P = 4.39×10^{-6}, unpaired t-test, two-tailed).

d, Western blot of LKB1 expression in WT, AMPKα KO, and LKB1-KO MEFs, representative of at least two experiments. Full blot is shown in **Source Data**.

e, (Left) Maximum ExRai AMPKAR ratio change in HeLa cells without (black, n = 27 cells from 3 experiments) or with (pink, n = 28 cells from 3 experiments) mCherry-LKB1 expression in response to 2-DG (40 mM; ****P = 3.01×10^{-7}, unpaired t-test, two-tailed). (right) Maximum ratio change of mito-ExRai AMPKAR in HeLa cells without (black, n = 15 cells from 3 experiments) or with (pink, n = 15 cells from 3 experiments) mCherry-LKB1 expression in response to 2-DG (40 mM; ns P = 0.25, unpaired t-test, two-tailed).

f, Western blot of candidate CaMKK2 KO MEFs generated via CRISPR/Cas9. GuideC was selected for further use. Representative of at least two trials. Full western blot is shown in **Source Data**.

g (left) Average response to 2-DG (40 mM) stimulation of cytoplasmic ExRai AMPKAR in WT (black, reproduced from **Fig 1e**) and CaMKK2 KO MEFs (pink, n = 13 cells from 6 experiments), along with maximum ratio changes (****P = 1.74×10^{-6}, unpaired t-test, two-tailed). (middle) Average response to 2-DG (40 mM) stimulation of mito-ExRai AMPKAR in WT (black, reproduced from **Fig 2c**) and CaMKK2 KO MEFs (pink, n = 12 cells from 6 experiments), along with maximum ratio changes (** P = 0.0015, unpaired t-test, two-tailed). (right) Average response to 2-DG (40 mM) stimulation of lyso-ExRai AMPKAR in WT (black, reproduced from **Fig 2b**) and CaMKK2 KO MEFs (pink, n = 7 cells from 4 experiments), along with maximum ratio changes (** P = 0.0013, unpaired t-test, two-tailed).

h, (Left) Maximum ratio change of cytoplasmic ExRai AMPKAR in HeLa cells without (black, n = 31 cells from 3 experiments) or with (pink, n = 29 cells from 3 experiments) mCherry-LKB1 expression in response to MK-8722 (500 nM; ****P = 3.91×10^{-14}, unpaired t-test, two-tailed). (Right) Maximum ratio change of mito-ExRai AMPKAR in HeLa cells without (black, n = 17 cells from 3 experiments) or with (pink, n = 24 cells from 3 experiments) mCherry-LKB1 expression in response to MK-8722 (500 nM; ns P = 0.056, unpaired t-test, two-tailed). Dot plots show the mean ± SEM.

Supplementary Figure 3. Nuclear AMPK activity is due to shuttling of AMPKα2 from the cytoplasm to the nucleus.

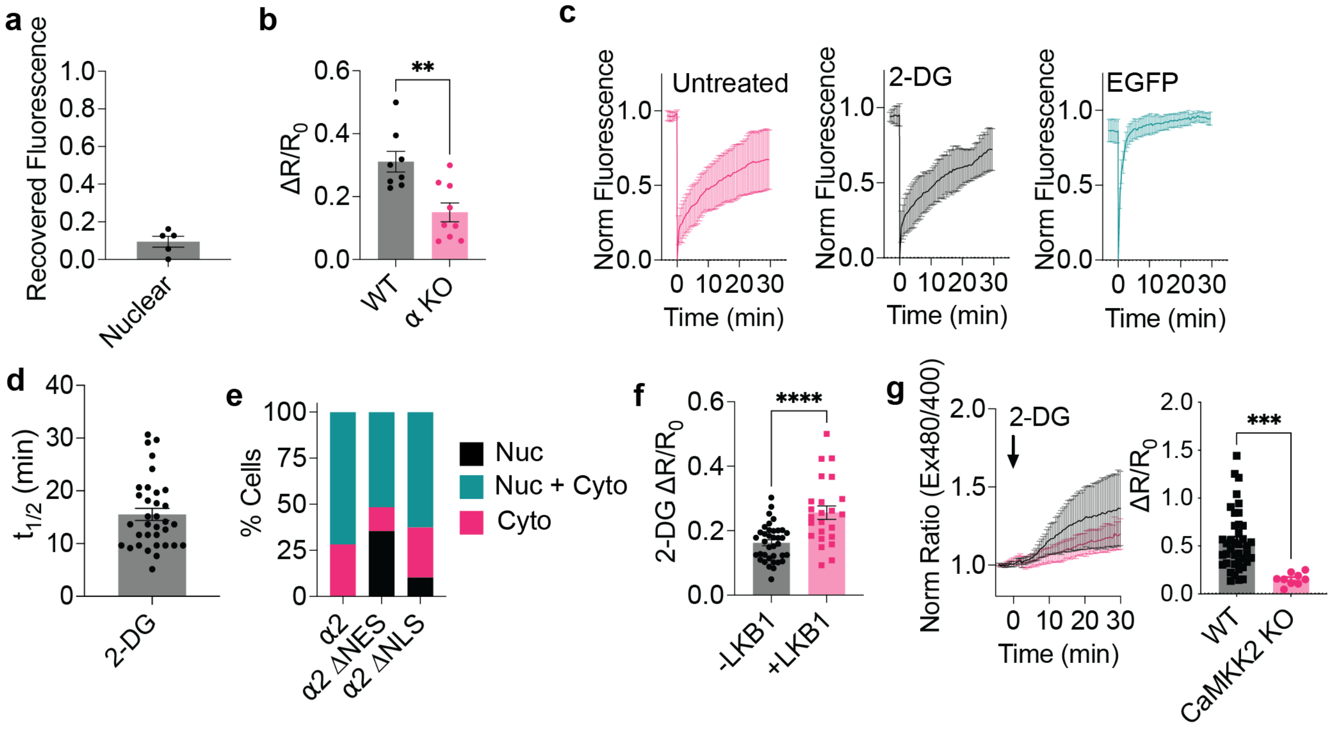

a, Percent fluorescence recovery after photobleaching (FRAP) of ExRai AMPKAR-NLS (n = 5 cells from 2 experiments).

b, AMPK activity in the nucleus following treatment with 10 mM 2-DG for 30 min in WT (black, n = 8 cells from 4 experiments) and AMPK KO MEFs (pink, n = 9 cells from 3 experiments; **P = 0.0026, unpaired t-test, two-tailed).

c, Average FRAP recovery curves of nuclear EGFP-AMPKα2 in AMPKα KO MEFs either untreated (pink, n = 9 cells from 3 experiments) or treated with 2-DG (black, 40 mM, n = 11 cells from 3 experiments) immediately before FRAP experiment began, as well as EGFP control (teal, n = 13 cells from 2 experiments).

d, Time to half-maximum of 2-DG-induced AMPK activity, analyzed from **Fig 4b**.

e, Quantification of mScarlet-AMPKα2, mScarlet-AMPKα2 ΔNES (L546A and L550A), mScarlet-AMPKα2 ΔNLS (K224A) localization to either only the nucleus (nuc), cytoplasm (cyto), or nucleus and cytoplasm (nuc + cyto). Representative of two independent experiments.

f, Average response of nuclear-localized ExRai AMPKAR in HeLa cells without (black; n =37 cells from 4 experiments) and with (pink; n = 24 cells from 4 experiments) mCherry-LKB1 expression and treated with 2-DG (40 mM) (****P = 2.19×10^{-5}, unpaired t-test, two-tailed).

g, Average response to 2-DG (40 mM) stimulation of cytoplasmic ExRai AMPKAR in WT (black, reproduced from **Fig 4b**) and CaMKK2 KO MEFs (pink, n = 9 cells from 3 experiments), along with maximum ratio changes (***P = 0.0006, unpaired t-test, two-tailed). Response curves show the mean ± SD, dot plots show the mean ± SEM.

Supplementary Table 1. Primers used for molecular cloning

Primer Number	Purpose	Sequence (5' to 3')
1	ExRai AMPKAR T/A, forward	GACGATAAGGATCCCATGAGGAGAGTGGCTGCTCTGGTGGATCTGGGC
2	ExRai AMPKAR T/A, reverse	CCGCCAGTGTGATGGATATCTGCAGAATTCCTAGCGATCAACTTTGTTCTGCTCGAGGCA
3	ExRai AMPKAR, forward	AAGGATCCCATGAGGAGAGTGGCTACCCTG
4	ExRai AMPKAR, reverse with stop codon	CCGCCAGTGTGATGGATATCTGCAGAATTCCTAGCGATCAACTTTGTTCTGCTCGAGGCA
5	ExRai AMPKAR, to remove stop codon forward	CAGAACAAAGTTGATCGCTATGAATTCCCCAAAAAGAAG
6	ExRai AMPKAR, to remove stop codon reverse	CTTCTTTTTGGGGAATTCATAGCGATCAACTTTGTTCTG
7	LAMP1, forward	GCGCAAGCTTGCGGCCGCCACCATGGCGGCGCCCGGCAGCGCCCGG
8	LAMP1, reverse	GCGCGGATCCGCACCACCGCCACCACCGATAGTCTGGTAGCCTGCG
9	mScarlet-AMPKa2, forward	TCAGATCCGCTAGCGCTACCGGTCGCCACCATGGTGAGCAAGGGCGAGGCAGTGATCAAG
10	mScarlet-AMPKa2, reverse	GCTTGAGCTCGAGATCTGAGTCCGGCCGGACTTGTACAGCTCGTCCATGCCGCCGGTGGA
11	3x NLS-mScarlet-AMPKa2, forward	AGAAAGGTAGGATCCAGTATGGTGAGCAAGGGCGAGGCAGTGATCAAG
12	3x NLS-mScarlet-AMPKa2, reverse	CTTGATCACTGCCTCGCCCTTGCTCACCATACTGGATCCTACCTTTCT
13	AMPKα2 ΔNLS	AAGCCCAAATCTTTAGCTGTGAAAGCCGCCAAGTGGCACCTTGGGATCCG
14	AMPKα2 ΔNES	GAAATGTGCGCCAGTGCAATCACTGCTGCAGCCCGTTGAGGATCC
